# Enhanced and controlled chromatin extraction from FFPE tissues and the application to ChIP-seq

**DOI:** 10.1186/s12864-019-5639-8

**Published:** 2019-03-29

**Authors:** Jian Zhong, Zhenqing Ye, Chad R. Clark, Samuel W. Lenz, Justin H. Nguyen, Huihuang Yan, Keith D. Robertson, Gianrico Farrugia, Zhiguo Zhang, Tamas Ordog, Jeong-Heon Lee

**Affiliations:** 1 Epigenomics Development Laboratory, Epigenomics Program, Center for Individualized Medicine, Mayo Clinic, Stabile building 12-04, 200 First Street SW, Rochester, MN 55905 USA; 2 Division of Biomedical Statistics and Informatics, Department of Health Science Research, Mayo Clinic, Rochester, MN 55905 USA; 3 Department of Transplant, Mayo Clinic, Jacksonville, FL 32224 USA; 4 Department of Biochemistry and Molecular Biology, Mayo Clinic, Rochester, MN 55905 USA; 5 Department of Molecular Pharmacology and Experimental Therapeutics, Mayo Clinic, Rochester, MN 55905 USA; 6 Epigenomics Program, Center for Individualized Medicine, Mayo Clinic, Rochester, MN 55905 USA; 7 Enteric Neuroscience Program, Mayo Clinic, Rochester, MN 55905 USA; 8 Department of Pediatrics and Department of Genetics and Development, Institute for Cancer Genetics, Columbia University, New York, NY 10032 USA; 9 Division of Gastroenterology and Hepatology, Department of Medicine, Mayo Clinic, Rochester, MN 55905 USA; 10 Department of Physiology and Biomedical Engineering, Mayo Clinic, Rochester, MN 55905 USA; 11 Division of Experimental Pathology and Laboratory Medicine, Department of Laboratory Medicine and Pathology, Mayo Clinic, Rochester, MN 55905 USA

**Keywords:** FFPE tissues, Chromatin extraction, ChIP-seq

## Abstract

**Background:**

Epigenetic dysregulation is involved in the etiology and progression of various human diseases. Formalin-fixed paraffin-embedded (FFPE) samples represent the gold standard for archiving pathology samples, and thus FFPE samples are a major resource of samples in clinical research. However, chromatin-based epigenetic assays in the clinical settings are limited to fresh or frozen samples, and are hampered by low chromatin yield in FFPE samples due to the lack of a reliable and efficient chromatin preparation method. Here, we introduce a new chromatin extraction method from FFPE tissues (Chrom-EX PE) for chromatin-based epigenetic assays.

**Results:**

During rehydration of FFPE tissues, applying a tissue-level cross-link reversal into the deparaffinized tissue at 65 °C dramatically increased chromatin yield in the soluble fraction. The resulting chromatin is compatible with targeted ChIP-qPCR and genome-wide ChIP-seq approaches. The chromatin prepared by Chrom-EX PE showed a gradual fragmentation pattern with varying incubation temperature. At temperatures below 37 °C, the majority of soluble chromatin is over 1 kb. The soluble chromatin prepared in the range of 45–60 °C showed a typical nucleosomal pattern. And the majority of chromatin prepared at 65 °C is close to mononucleosomal size. These observations indicate that chromatin preparation from FFPE samples can be controlled for downstream chromatin-based epigenetic assays.

**Conclusions:**

This study provided a new method that achieves efficient extraction of high-quality chromatin suitable for chromatin-based epigenetic assays with less damage on chromatin. This approach may provide a way to circumvent the over-fixed nature of FFPE tissues for future technology development.

**Electronic supplementary material:**

The online version of this article (10.1186/s12864-019-5639-8) contains supplementary material, which is available to authorized users.

## Background

Epigenetic alterations have been well described in the pathogenesis of various human diseases [1–3]. In combination with next-generation sequencing technologies, genome-wide epigenetic profiling has provided an unprecedented opportunity to understand the regulatory mechanisms underlying genome activities in disease etiology and progression [3]. Epigenetic methodologies are well established in cultured cells but are still limited in clinical samples. FFPE samples are the gold standard for archiving patient samples and have been utilized in the diagnosis and clinical management of the vast majority of diseases [4]. While FFPE methods include treatment with formaldehyde, it is often applied at a higher concentration of formalin and for a longer time to ensure proper and longer preservation of the relatively large pieces of tissue samples for downstream applications such as immunohistochemistry. In these cases, FFPE samples may be heavily over-fixed (i.e., more heavily cross-linked), which makes it challenging to obtain chromatin with high yield for common epigenetic assays like chromatin immunoprecipitation (ChIP) or other chromatin-based assays including chromatin accessibility, nucleosome positioning, and chromatin-chromatin interaction. Over-fixation in FFPE samples interferes with chromatin analysis in different ways. First, over-fixation necessitates the use of harsher chromatin fragmentation methods which, in turn, damages the chromatin. Harsher chromatin isolation approaches may increase the chromatin yield marginally, but these small gains are usually negated by loss of chromatin integrity. Second, over-fixation may manifest in random crosslinking of chromatin with other cellular components resulting in low signal-to-noise ratio and very low chromatin yields. There have been some advances in chromatin extraction methods for FFPE samples [5–7], but a reliable and efficient chromatin preparation method is still needed.

The basic repeating unit of chromatin is nucleosome, and it is associated with proteins and RNAs. Nucleosomes and chromatin include epigenetic information such as DNA-based epigenetic marks, histone tail modifications, association with transcription factors or chromatin proteins or regulatory RNAs, nucleosome positioning, accessibility, and chromatin-chromatin interaction. While chromatin-based epigenetic assays are feasible in FFPE tissues [5–9], the detailed profiles of extracted chromatin are limited and not well demonstrated. The first chromatin extraction method from FFPE tissues called pathology tissue-ChIP (PAT-ChIP) was established and validated for ChIP assay using a combination of micrococcal nuclease (MNase) digestion and extensive sonication in the presence of high concentration of SDS [5]. Another chromatin extraction method called fixed-tissue chromatin immunoprecipitation sequencing (FiT-seq) was developed by introducing 1 h heating at 40 °C, proteinase K treatment, and extensive sonication to improve chromatin extraction [6]. The chromatin isolated from FiT-seq approach was validated for ChIP assay but the information about chromatin yield and the sizes of extracted chromatin were not assessed. In addition to the ChIP assay, one group demonstrated the feasibility of chromatin accessibility assay in FFPE tissues using DNase-seq [10]. Currently, nucleosome positioning assay [11] and the methodologies dependent on chromatin integrity such as chromatin interaction assays [12] are not applicable to FFPE tissues. We hypothesized that regaining chromatin integrity close to intact or at least recovering it partially by reducing cross-links before chromatin extraction may be beneficial to achieve better chromatin yield and quality to overcome heavily cross-linked nature of FFPE tissues.

In this study, we introduced a novel chromatin extraction method from FFPE tissues called Chrom-EX PE technology to increase soluble chromatin yield with the possibility of controlled preparation of chromatin from FFPE tissues. This approach includes a tissue-level, cross-link reversal step applied to deparaffinized tissue to overcome the disadvantages of over-fixed chromatin. We achieved approximately 70–90% soluble chromatin yields from mouse FFPE tissues, and the isolated chromatin was compatible with ChIP-seq for histone mark and chromatin-associated protein. Furthermore, the chromatin prepared by Chrom-EX PE showed a gradual fragmentation pattern with varying incubation temperatures. We believe this approach can be used to obtain high quality chromatin for ChIP-seq without exposing the sample to harsh treatment, and is potentially applicable to technology development for other chromatin-based epigenetic assays.

## Results

### Tissue-level cross-link reversal dramatically increases soluble chromatin from FFPE tissues

**Fig. 1:**
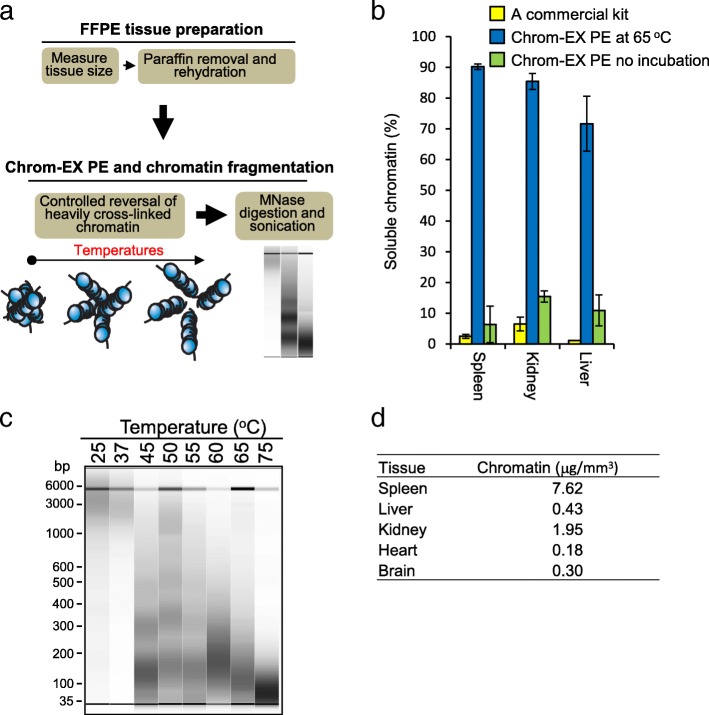
A tissue-level cross-link reversal allows efficient extraction of high-quality chromatin from FFPE tissues. **a** Schematic diagram of chromatin extraction method from FFPE tissues (ChromEX-PE). A tissue-level, cross-link reversal was introduced by incubating deparaffinized tissue in the range of temperature from 25 to 75 °C before chromatin extraction. **b** Chrom-EX PE dramatically increases soluble chromatin from FFPE tissues. Chromatin was prepared by Chrom-EX PE with 65 °C overnight incubation or without incubation. A commercial kit from Active motif was used as control and for comparison. DNAs were purified from soluble fraction and insoluble pellet fraction and were quantified using Qubit dsDNA High Sensitivity assay. The percentage of soluble chromatin was calculated from mouse spleen, kidney, and liver FFPE tissues. The data were generated from two independent experiments. **c** Chrom-EX PE generates different sizes of chromatin in a controlled manner. Chromatin was prepared from mouse liver FFPE tissues by Chrom-EX PE in the range of temperatures from 25 °C to 75 °C. Deparaffinized tissue was incubated in the indicated temperature overnight in the chromatin stabilization buffer. In the range of 25–37 °C, a majority of soluble chromatin is larger than 1 kb. The temperature ranges (45–55 °C) produce nucleosomal DNA patterns. The majority of DNA was mononucleosomal DNA at 60 °C incubation and DNA size is closer or smaller than mononucleosomal DNA with temperature above 60 °C. **d** Chromatin yield by Chrom-EX PE from various mouse tissues. The tissue volume is measured in FFPE block and two 20-μm sections were processed by Chrom-EX PE at 65 °C condition with chromatin stabilization buffer. The DNA amount purified from soluble fraction was measured and the yield was calculated per tissue volume

The major challenge for chromatin extraction from FFPE tissues is low chromatin yield due to high cross-linking level of the tissue sample during preparation and long storage time of FFPE samples. We hypothesized reducing cross-linking before chromatin preparation would increase chromatin yield from FFPE tissues. To test this hypothesis, we introduced a tissue-level cross-link reversal step by heating before chromatin preparation called Chrom-EXPE (Fig. 1a). As incubation of cross-linked chromatin at 65 °C is routinely utilized in ChIP assay, we first tested tissue-level cross-link reversal with overnight incubation at 65 °C. As a control and for comparison, we tested a commercial kit (Active Motif) for chromatin extraction from FFPE tissues following the manufacturer’s instructions. After tissue-level cross-link reversal, MNase digestion and sonication was utilized to extract chromatin. Notably, the insoluble pellet after centrifugation is much smaller with samples processed with tissue-level cross-link reversal compared with the samples processed with the commercial kit or without overnight incubation. The soluble chromatin yield was calculated from purified DNA from soluble and insoluble pellet fractions after extensive cross-link reversal as described in conventional ChIP protocols. Surprisingly, tissue-level cross-link reversal dramatically increased chromatin yield in FFPE samples from mouse spleen, kidney, and liver (Fig. 1b). The soluble chromatin yields achieved 71.6, 85.4 and 90.2% for liver, kidney, and spleen, respectively, by Chrom-EX PE, while the soluble chromatin yields reached 1.15, 6.48 and 2.54% with the Active Motif commercial kit and 6.37, 15.45, 10.95% without 65 °C incubation for the same tissues. These results demonstrate that Chrom-EX PE increases the effectiveness of chromatin preparation from FFPE samples and can achieve chromatin yields up to 90% of the yields typically obtained using conventional techniques from fresh or frozen tissues.

To further investigate chromatin yields in various incubation temperatures, we performed the experiment at four different temperatures (25 °C, 45 °C, 55 °C, and 65 °C) using FFPE samples from liver tissue. To accurately measure the impacts of incubation temperature on chromatin yield, we isolated the chromatin by sonication but no MNase treatment after tissue-level cross-linking reversal. The yield of soluble chromatin gradually increases with temperature in 45 – 65 °C range compared with the yield from room temperature (Additional file 1), suggesting different level of cross-linking reversal contributes to the increase of soluble chromatin.

### Chrom-EX PE enables chromatin to be prepared in a controlled manner

We checked chromatin patterns generated in different incubation temperatures for tissue-level cross-link reversal. Chromatin was prepared from mouse liver FFPE tissues by Chrom-EX PE in the range of 25 to 75 °C, and DNA was purified from the resulting soluble chromatin and analyzed by the Fragment Analyzer (Fig. 1c). In general, low temperatures generated larger DNA fragments over 1 kb and DNA sizes gradually decreased to around mononucleosomal size with the increasing incubation temperatures. In the range of 25–37 °C, a majority of soluble chromatin is larger than 1 kb and a very small fraction of DNA fragments around 50–100 bp. The temperature range of 45–55 °C produced a nucleosomal DNA profile that is typically observed when frozen tissues and cell lines are treated with MNase. The majority of DNA was mononucleosomal size in the range of 60–65 °C, and DNA size is smaller than mononucleosomal DNA at 75 °C. These observations indicated that Chrom-EX PE is able to prepare chromatin in a controlled manner from FFPE tissues. It is noteworthy that the sizes of most soluble chromatin in the range of 45–65 °C are about 100–500 bp, further increasing usable chromatin in the assays and well suited for the requirement of next-generation sequencing library preparation for genome-wide chromatin-based epigenetic assays.

### Chromatin yields from mouse FFPE tissues by Chrom-EX PE

The overall experimental quality of chromatin-based epigenetic assays is typically dependent on the total cellularity but not the weight of tissue sample. As tissues show different cellularity, we next sought to address chromatin yield from different FFPE tissues by Chrom-EX PE in the optimized chromatin stabilization buffer. The tissue volume is measured in the FFPE block, and two 20 μm sections were processed by Chrom-EX PE at 65 °C condition. The DNA amount purified from soluble fraction was measured and the yield was calculated (Fig. 1d).

### Chromatin generated by Chrom-EX PE is compatible with ChIP assays

**Fig. 2:**
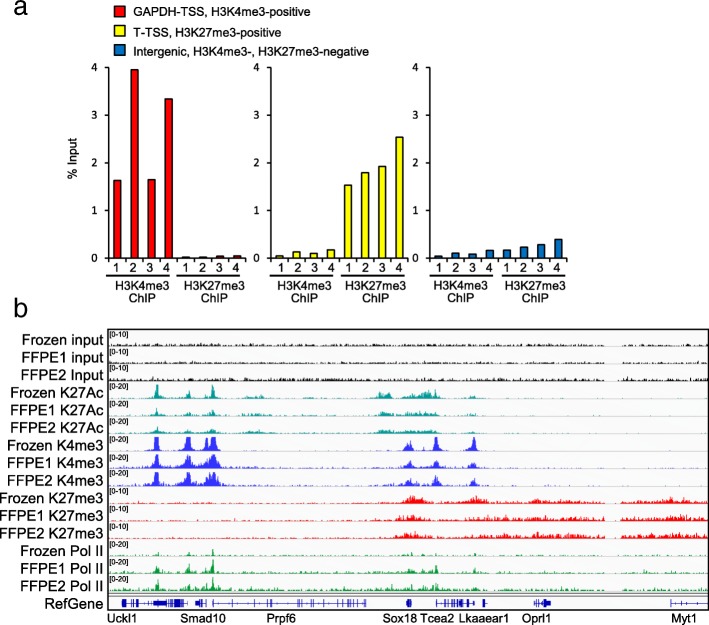
The chromatin generated by Chrom-EX PE is compatible with ChIP-qPCR and ChIP-seq. **a** The chromatin generated by Chrom-EX PE produced consistent and high enrichment in ChIP-qPCR approach. Two 20-μm sections from mouse liver FFPE tissues were processed by Chrom-EX PE at 65 °C condition. The resulting chromatin was immunoprecipitated as described in the Materials and Methods. ChIP DNAs were analyzed by qPCR and enrichment in the tested loci is shown as the percentage of input. Four individual mouse FFPE liver tissues were used in experiment. The genomic location of each primer pair along with peak profile of H3K4me3 and H3K27me3 marks are shown in the Additional file 3. **b** Chromatin generated by Chrom-EX PE produced comparable ChIP-seq peaks with the results from frozen tissues. ChIP-seq experiment was performed in the chromatin generated by Chrom-EX PE at 65 °C condition from two 20-μm sections of mouse liver FFPE tissues. As controls, ChIP-seq data was generated from frozen tissues as described in the Materials and Methods. Snapshot image are shown for 3 histone marks (H3K27ac, K27 ac; H3K4me3, K4me3; H3K27me3, K27me3) and RNA polymerase II (Pol II). ChIP-seq results were visualized in a 218 kb genomic region using the Integrative Genomics Viewer [17, 18]. FFPE 1 and Frozen are the pair originated from the same mouse

To investigate whether the chromatin generated by Chrom-EX PE is compatible with chromatin-based epigenetic assays, we elected to use chromatin immunoprecipitation assay, which is the most often used methodology in chromatin-based epigenetic analyses. The chromatin prepared by Chrom-EX PE at 65 °C from four different mouse liver FFPE tissue was incubated with anti-H3K4me3 or anti-H3K27me3 antibodies and further processed following published methods [13, 14]. The ChIP products were analyzed by real-time PCR in a transcriptionally active region (GAPDH-TSS, positive for H3K4me3), in a developmentally repressed region (T-TSS, positive for H3K27me3), and in an intergenic region (negative for both H3K4me3 and H3K27me3) (Fig. 2a and Additional file 2). The enrichment of H3K4me3 mark is observed only in transcriptionally active TSS, and H3K27me3 mark is observed only in developmentally repressed locus in all four FFPE tissues. The DNA size of chromatin input from 4 FFPE samples is in the range of 100–200 bp without DNAs larger than 1 kb (Additional file 3), consistent with the previous observation (Fig. 1c) and showing consistency of the Chrom-EX PE approach. These results demonstrate that the chromatin prepared from FFPE tissues using Chrom-EXPE is compatible with a targeted ChIP-qPCR approach. Next, we further investigated the compatibility of Chrom-EX PE with next-generation sequencing library preparation for genome-wide ChIP-seq. The chromatin input isolated by Chrom-EX PE from mouse liver FFPE tissue was subjected to ChIP for histone marks (H3K27Ac and H3K4me3, H3K27me3) and a chromatin-associated protein (RNA Polymerase II, Pol II). To compare the performance of Chrom-EX PE, we also generated ChIP-seq data from frozen tissues originating from the same mouse using published ChIP-seq protocol [13, 14]. To evaluate the reproducibility of Chrom-EX PE, we performed the experiment in two replicates. The purified ChIP DNA was subjected to library preparation as published [13] and the library DNAs were analyzed by the Fragment Analyzer (Additional file 4). The libraries were sequenced on Illumina HiSeq 2000 platform. Peaks were called using the MACS2 algorithm [15] for H3K4me3 and H3K27Ac and SICER [16] for H3K27me3 and RNA Pol II at FDR < =1%. The difference of overall enrichment is variable in sample types per mark (Additional file 5). However, peak patterns visualized in the Integrative Genome Viewer [17, 18] were highly similar among the datasets from FFPE liver tissue by Chrom-EX PE and those from frozen liver tissues by published ChIP-seq protocol (Fig. 2b). As expected [19, 20], H3K4me3 and H3K27Ac peaks are primarily located at TSS of active genes, whereas H3K27me3 peaks are distributed over PRC2-repressed genes such as MYT1. These results strongly suggest that the chromatin generated by Chrom-EX PE technology is compatible with ChIP-seq approach.

### Highly correlated and consistent ChIP-seq data are generated from FFPE tissue by Chrom-EX PE

The ChIP-seq data for each mark was generated from two 20-μm FFPE-tissue sections from mouse liver and spleen using Chrom-EX PE technology. To compare the performance of Chrom-EX PE, we also generated ChIP-seq data from frozen tissues originating from the same mouse. The mapping details of all ChIP-seq libraries are summarized (Additional file 6) [21]. In general, we did not observe clear differences in % uniquely mapped pairs, library complexity, and % duplicates between libraries generated from FFPE and frozen tissues. Some libraries generated by Chrom-EX PE showed higher % duplicate, low complexity, and low % unique pairs but other libraries generated in the independent experiment showed the expected range of QC results. We believe higher % duplicate, low complexity, and low % unique pairs in some of ChIP-seq data observed in FFPE samples is caused by over-amplification of the library [22] but not by Chrom-EX PE technology. Importantly, the total peak numbers for these libraries still achieve the requirement for the data analysis.

**Fig. 3:**
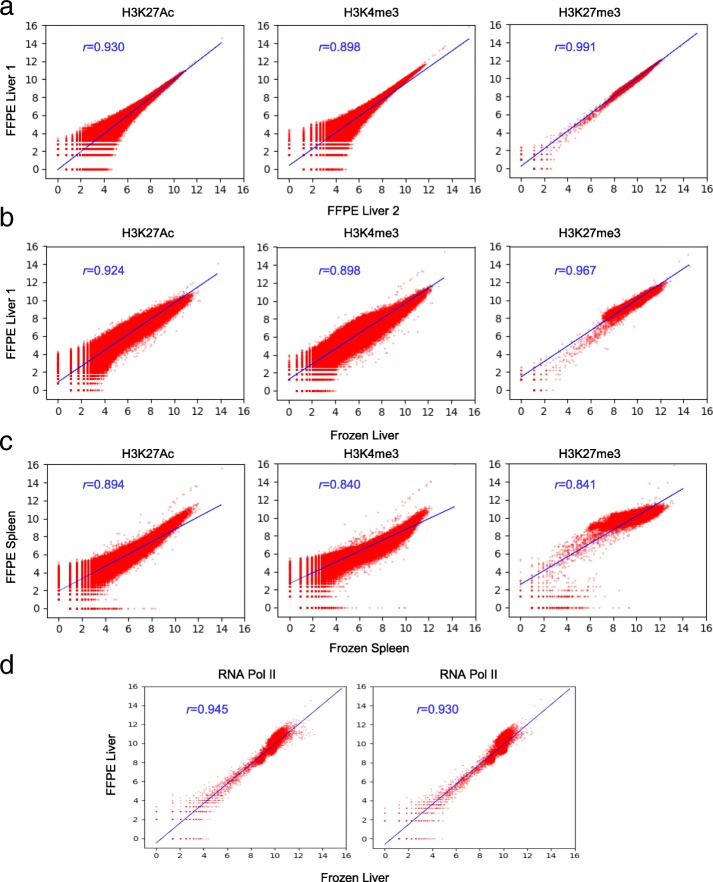
Highly correlated genome-wide ChIP-seq data are generated from mouse FFPE tissues by Chrom-EX PE. **a** Scatter plots showing the correlation between ChIP-seq datasets generated from the indicated repeats for FFPE liver tissue with H3K27Ac, H3K4me3 and H3K27me3 marks. The genome was divided into bins of 5 kb for H3K4me3 and H3K27Ac marks and 100 kb for H3K27me3 mark, and the number of mapped reads in the individual bins was calculated. *r*, Pearson correlation coefficient. **b** Scatter plots showing the correlation between ChIP-seq datasets generated from FFPE liver tissue by Chrom-EX PE and the corresponding frozen liver tissues by conventional ChIP-seq for H3K27Ac, H3K4me3 and H3K27me3 marks. **c** Scatter plots showing the correlation between ChIP-seq datasets generated from a pair of mouse spleen tissues. **d** Scatter plots showing the correlation between ChIP-seq datasets generated from FFPE liver tissue by Chrom-EX PE and the corresponding frozen liver tissues by conventional ChIP-seq for RNA polymerase II

To investigate the experimental consistency of Chrom-EX PE technology, we performed Pearson correlation analysis between the datasets generated by Chrom-EX PE from experimental replicates. We observed uniformly high correlation for three histone marks with Pearson correlation coefficients ranging between 0.898 and 0.991 (*p* < 0.001) (Fig. 3a). The detailed peak numbers and overlapping rate in experimental replicates are shown for FFPE samples (Table 1) and for frozen tissues (Additional file 7). We calculated the overlap rate based on the dataset with fewer peaks to investigate how many peaks are observed in the same locations in two replicates. For H3K27Ac mark, about 92,000 peaks were detected with 91% overlap rate in repeats. About 48,000 peaks were detected with 84% overlap rate for H3K4me3 mark and about 20,000 peaks were detected with 64% overlap for H3K27me3 mark. These results indicate that ChIP-seq data generated by Chrom-EX PE is highly reproducible and consistent.

**Table 1:** Peak numbers and overlap rate of peaks from experimental repeats from FFPE samples

Mark	Total Peaks 1	Total Peaks 2	% Overlap
H3K27ac	128,624	92,165	90.91%
H3K4me3	56,688	48,129	83.90%
H3K27me3	19,765	23,234	64.14%

We further investigated whether ChIP-seq dataset generated by Chrom-EX PE is comparable with datasets generated from frozen tissues using a published ChIP-seq protocol. Pearson correlation analysis show uniformly high correlation for three histone marks between datasets generated from FFPE tissues and frozen tissues obtained from liver (Fig. 3b) and spleen (Fig. 3c). Pearson correlation coefficients range between 0.898 and 0.967 (*p* < 0.001) in liver tissue and between 0.840 and 0.894 (*p* < 0.001) in spleen tissue. The detailed peak numbers and overlap rate in FFPE and frozen tissues are shown in Table 2. In general, the overall peak number detected is comparable and overlap rate of peak is in the range of 61–82% in the liver tissue and in the range of 82–95% in the spleen tissue. For example, 128,000 H3K27Ac peaks were detected from FFPE liver tissue by Chrom-EX PE and 113,000 peaks were detected from frozen liver tissue with 71% overlap rate. 56,000 H3K4me3 peaks were detected from FFPE liver tissue and 49,000 peaks were detected from frozen liver tissue with 61% overlap rate. 19,000 H3K27me3 peaks were detected from FFPE liver tissues and 30,000 peaks were detected from frozen liver tissue with 82% overlap rate. These results indicate a high degree of similarity among ChIP-seq dataset generated from FFPE tissue by Chrom-EX PE and frozen tissue by conventional ChIP-seq.

**Table 2:** Peak numbers and overlap rate of peaks from FFPE and frozen tissue pairs

Mark	Total Peaks in Frozen	Total Peaks in FFPE1	% Overlap
m_liver H3K27ac	113,364	128,624	71.08%
m_liver H3K4me3	49,441	56,688	61.24%
m_liver H3K27me3	30,193	19,765	82.21%
m_spleen H3K27ac	98,641	71,648	82.40%
m_spleen H3K4me3	52,991	33,512	94.93%
m_spleen H3K27me3	38,955	13,332	85.85%
m_liver Pol II	10,843	12,825	83.65%
m_liver Pol II	7874	16,421	92.01%

To determine whether Chrom-EX PE technology is applicable to large and non-histone protein or chromatin complexes, we performed ChIP-seq for RNA polymerase II (RNAP II) in two pairs of FFPE and frozen mouse liver tissues. As expected, RNA Pol II peaks shown in the IGV browser overlap with H3K4me3 marks in TSSs of actively transcribed genes (Fig. 2b), and peak patterns are similar among the datasets generated from FFPE and frozen liver tissues. Pearson correlation coefficients range from 0.930 to 0.945 (*p* < 0.001) in matched sample pairs (Fig. 3d). The detailed peak numbers and overlapping rate in match pairs are shown in Table 2. About 12,000 and 16,000 RNA Pol II peaks were detected in two different FFPE tissues. These results indicate that Chrom-EX PE is applicable to at least some non-histone protein or chromatin complex in archived FFPE tissues.

## Discussion

Fixation of cells by formaldehyde was introduced in the ChIP assay to increase the stability of interactions in chromatin by making covalent bonds between neighboring amino groups. Consequently, the solubility of chromatin is much lower in fixed cells compared with unfixed cells. Over-fixation in FFPE tissues is believed to be detrimental to chromatin extraction due to the nature of heavily cross-linked chromatin. Harsh sonication was typically introduced to overcome low solubility but this introduces adverse damage in chromatin. We introduced a tissue-level cross-linking reversal before chromatin preparation called Chrom-EX PE. This approach dramatically increases soluble chromatin from FFPE tissue and allows us to produce various degrees of chromatin showing a gradual shift from partial, nucleosomal pattern, to smaller than a mononucleosome after MNase digestion and sonication. We validated here that the chromatin generated by Chrom-EX PE at 65 °C is compatible with the ChIP assay. The chromatin generated in the range of 45–55 °C showed a nucleosomal pattern that is typically produced from frozen tissues and cell lines, and may be compatible with nucleosome positioning along with ChIP assay (Fig. 1c). Partial digestion patterns at lower temperatures may be compatible with mapping of chromatin accessibility. Further research is required to investigate whether Chrom-EX PE technology is applicable to nucleosome positioning, chromatin accessibility, or chromatin-chromatin interaction assays.

Based on chromatin yield and fragmentation pattern, we believe that incubation of FFPE tissue in chromatin stability buffer will cause decross-linking of covalent bonds and the kinetics of decross-linking is dependent on temperature and time. In the range of incubation temperatures around 37–55 °C, decross-linking reaction may be slow but more closely recover the original chromatin structure. At higher temperature, the reaction may be faster and more effective. Published chromatin preparation methods [6–8] support this hypothesis. FiT-seq incubated FFPE samples at 40 °C for 1 h in 0.1% SDS buffer [6]. We did not test 40 °C in this study but 40 °C may induce some degree of cross-link reversal and subsequently increase soluble chromatin yield. With the report of DNase-seq in FFPE samples [10], we noticed that DNase I treatment was applied after incubation of deparaffinized tissues in 37 °C for 2 h for FFPE samples but not for fresh cells. Incubation at 37 °C may also induce some degree of decross-link to allow DNase I accessibility to regions of genome. Consistently, our result showed that a majority of chromatin is larger than 1 kb and a small fraction of DNA fragments around 50–100 bp is observed at 37 °C incubation (Fig. 1c). These observations indicate that controlled heat treatment of the FFPE sample may reduce the extent of cross-linking and render chromatin fragmentation and extraction more effective in downstream processing. As typically done in cell line and frozen tissue, chromatin fragmentation and extraction in Chrom-EX PE technology can be done by MNase, sonication, or combined MNase and sonication depending on the purpose of downstream experiment. These suggest that some degree of cross-link reversal by heat treatment may be a way to overcome the heavily cross-linked nature of FFPE samples. Further investigation is required to understand the close relationship between decross-linking kinetics and recovery of chromatin structure in different incubation conditions. We believe Chrom-EX PE technology will provide an opportunity to improve the existing epigenetic technologies and more desirable outcomes for heavily cross-linked FFPE samples.

## Conclusions

This study provided a new Chrom-EX PE technology that achieves efficient extraction of high-quality chromatin from FFPE tissues. The chromatin generated by this technology shows a gradual fragmentation pattern dependent on temperature. We determined that the chromatin generated at 65 °C is compatible with ChIP assay in targeted and genome-wide approaches. Importantly, the chromatin generated at other temperatures may be suitable for chromatin-based epigenetic assays including accessibility, nucleosome positioning, and chromatin-chromatin interaction. This new technology will provide a better opportunity to perform chromatin-based epigenetic studies in archived FFPE samples.

## Methods

### Animals and tissues

Frozen mouse tissues were collected from healthy mice under the Mayo Clinic IRCUC A00001233–16. The tissues were excised and divided. One piece from each pair was flash-frozen in liquid nitrogen and stored at − 80 °C and the other was fixed in 20-fold excess volume of 10% neutral phosphate-buffered formalin (Leica Biosystems, Winnipeg, MB) for 48 h. Fixed tissue was dehydrated and embedded in paraffin using an automated tissue processor. FFPE blocks were stored at RT for at least 2 years. These samples were used to establish and optimize Chrom-EX PE protocol.

### Development of Chrom-EX PE, chromatin isolation from FFPE tissues

Two 20-μm sections from the FFPE blocks derived from mouse tissues were deparaffinized with xylene substitute and were rehydrated by progressively increasing percentage of water (95/5, 70/30, 50/50, 20/80). During the progressive dehydration with different ethanol concentrations, the tissues were resuspended in 0.5 ml of chromatin stabilization buffer (10 mM Tris-HCl, pH 7.5, 10 mM NaCl, 10 mM EDTA, 0.5% Triton X-100, 0.1% Sodium deoxycholate, 20% EtOH, proteinase inhibitor cocktails) and incubated in different temperatures ranging 25 °C to 75 °C overnight. As a control, the tissues were processed without overnight incubation. After the centrifugation (21,130 x g for 5 min), the supernatant was removed. And 0.25 ml of cell lysis buffer (10 mM Tris HCl, pH 7.5, 10 mM NaCl, 0.5% IGEPAL) was added and incubated on ice for 10 min. The lysates were washed with MNase digestion buffer (20 mM Tris-HCl, pH 7.5, 15 mM NaCl, 60 mM KCl, 1 mM CaCl_2_) and were incubated in the fresh 150 μL MNase digestion buffer containing proteinase inhibitor cocktails in the presence of 10 gel units of MNase (NEB, Cat.# M0247S) at 37 °C for 20 min with continuous mixing in thermal mixer. After adding the same volume of sonication buffer (100 mM Tris-HCl, pH 8.1, 20 mM EDTA, 200 mM NaCl, 2% Triton X-100, 0.2% sodium deoxycholate), the lysates were sonicated for 15 min (30 s on / 30 s off) using Bioruptor Twin (UCD-400) (Diagenode, Inc., Denville, NJ) and centrifuged at 21,130 x g for 10 min. The supernatant was transferred in a new tube. The pellet was resuspended in 100 ul of 1X FFPE Stop buffer 1 (50 mM Tris-HCl, pH 8.1, 10 mM EDTA, 100 mM NaCl, 1% Triton X-100, 0.1% Sodium deoxycholate, 0.05% SDS) and was subjected to sonication for 15 min (30 s on / 30 s off), and centrifuged at 21,130 x g for 10 min. The supernatant was collected into the previously saved fraction. The pellet was resuspended in 100 ul of 1X FFPE Stop buffer 2 (50 mM Tris-HCl, pH 8.1, 10 mM EDTA, 100 mM NaCl, 1% Triton X-100, 0.1% Sodium deoxycholate, 0.1% SDS) and was subjected to sonication for 15 min (30 s on / 30 s off), and centrifuged at 21,130 x g for 10 min. The supernatant was collected into the previously saved fraction and the combined soluble fraction was served as soluble chromatin. The equal volume of soluble chromatin was mixed with 2X ChIP elution buffer (20 mM Tris-HCl, pH 8.0, 20 mM EDTA, 300 mM NaCl, 10 mM DTT, 2% SDS) and incubated at 65 °C overnight. The pellet was resuspended in 1X ChIP elution buffer and incubated at 65 °C overnight. DNAs were purified using Min-Elute PCR purification kit after the treatment of RNase A and proteinase K. DNA amount was measured by the Qubit dsDNA High Sensitivity assay (Invitrogen, Q32851), and DNA size was analyzed by the Fragment Analyzer (Advanced Analytical Technologies; AATI; Ankeny, IA) using the High Sensitivity NGS Fragment Analysis Kit (Cat. #DNF-486). For the comparison of soluble chromatin yield, the ChIP-IT® FFPE Chromatin Preparation Kit (Active Motif, Cat # 53030) was utilized according to the manufacture’s instructions.

### Determination of chromatin yield from mouse FFPE tissues

The tissue image in FFPE block was captured every 5 cut, and the surface area of tissue was calculated by the NIH Image J program to determine the tissue size in the cuts. Two 20-μm sections were deparaffinized with xylene substitute and were rehydrated by progressively increasing percentage of water in ethanol up to 20%. The tissues were resuspended in 0.5 ml of chromatin stabilization buffer and incubated at 65 °C overnight. The tissues were processed as described above. Purified DNA was measured by the Qubit assay. The chromatin amount from each FFPE tissue is calculated by DNA amount per tissue size.

### ChIP-seq

Frozen tissues (25 mg) were homogenized for 30 s in PBS using tissue grinder (ACTGene, ACT-AG 3080). Homogenized tissues were cross-linked to final 1% formaldehyde, quenched with 125 mM glycine, and washed with TBS. The fixed homogenates were resuspended in cell lysis buffer and incubated on ice for 10 min. The lysates were washed with MNase digestion buffer and were incubated in the fresh 500 μL MNase digestion buffer containing proteinase inhibitor cocktails in the presence of 1000 gel units of MNase at 37 °C for 20 min with continuous mixing in thermal mixer. After adding the same volume of sonication buffer, the lysates were sonicated for 15 min (30 s on / 30 s off) using Bioruptor Twin and centrifuged at 21,130 x g for 10 min. The supernatants were served as chromatin input. For FFPE tissues, chromatin input was prepared from two 20-μm sections as described in the development of Chrom-EX PE. The chromatin input was incubated with anti-H3K4me3 (abcam ab8580, lot GR188707–1), anti- RNA pol II antibodies (Bethyl A300–653, lot 3), anti-H3K27Ac (CST 8173 BC) or anti-H3K27me3 (CST, 9733 s, lot 8) overnight. After adding 10–30 μL of protein G-magnetic beads, the reactions were further incubated for 3 h. The beads were extensively washed with ChIP buffer (50 mM Tris-HCl, pH 8.1, 10 mM EDTA, 100 mM NaCl, 1% Triton X-100, 0.1% sodium deoxycholate), high salt buffer (50 mM Tris-HCl, pH 8.1, 10 mM EDTA, 500 mM NaCl, 1% Triton X-100, 0.1% sodium deoxycholate), LiCl_2_ buffer (10 mM Tris-HCl, pH 8.0, 0.25 M LiCl_2_, 0.5% NP-40, 0.5% Sodium deoxycholate, 1 mM EDTA), and TE buffer. Bound chromatins were eluted and reverse-crosslinked at 65 °C overnight. DNAs were purified using Min-Elute PCR purification kit after the treatment of RNase A and proteinase K. ChIP enrichment was validated by performing qPCR in the genomic loci targeting the transcription start sites of an active or inactive gene and an intergenic region. ChIP-seq libraries were prepared using the Ovation ultralow DR Multiplex kit (NuGEN, San Carlos, CA) or the ThruPLEX® DNA-seq Kit V2 (Rubicon Genomics, Ann Arbor, MI) according to the manufacture’s instructions. The ChIP-seq libraries were sequenced to 51 base pairs from both ends on an Illumina HiSeq 2000 instrument in the Mayo Clinic Center for Individualized Medicine Medical Genomics Facility.

### Real-time PCR analysis

Real-time PCR analysis was performed using SYBR Green universal PCR mixes (Bio-Rad). The following primer sequences were used in the experiments: H3K4me3- and RNA pol II-positive control locus: mGAPDH-F: 5′- CTCATCCCCGCAAAGGCGGA -3′, mGAPDH-R: 5′- TCGGACCTGGCGATGGCTCG-3′. H3K27me3-positive control locus: mT1-F: 5′- GAGACGCCGATCCGCCGAAG -3′, mT1-R: 5′- ACTCTCCACTCCCACGCGCT-3′. H3K4me3-, RNA pol II-, and H3K27me3-negative control locus: mIntergenic-F: 5′-CCTGCTGCCTTGTCTCTCTC -3′, mIntergenic-R: 5′-ATGGCCTAGGGATTCCAGCA -3′.

### Mapping and analysis of ChIP-seq data

Raw sequencing reads were processed and analyzed using the HiChIP pipeline [21] to obtain visualization files and a list of peaks. Briefly, paired-end reads were mapped to the mouse reference genome (mm10) by BWA [23] with default settings, and only pairs with at least one of the ends being uniquely mapped were retained for further analysis. Duplicates were removed using the Picard tool set. (https://broadinstitute.github.io/picard/). Peaks were called using the MACS2 algorithm and SICER at FDR < 1%. Fragment size was calculated from properly mapped read pairs. Pearson correlation analysis was performed by our in-house scripts where all datasets were randomly downsized to 25 million pairs of reads. In brief, the whole genome was divided into 5-kb bins for H3K4me3 and H3K27Ac and 100-kb bins for H3K27me3 and RNA Pol II, and the number of mapped reads in each bin was calculated, in which genomic regions that have zero or missing values in all samples are excluded. The read count per bin was normalized to 25 million mapped reads (at least one end uniquely mapped and duplicates removed), or to 25 million mapped reads from non-peak regions (total - reads mapped to peak regions). The normalized counts by logarithm log2 (normalized count + 1) were used for pairwise correlation analysis with Pearson coefficient. Here, 1 is a pseudo-count to avoid an undefined error of logarithm of zero. We also profiled the average signal levels across peak center +/− 2 kb (H3K4me3 and H3K27Ac) and peak region +/− 2 kb (H3K27me3 and RNAPII) using ngs.plot [24]. For each pair of samples, peaks from the two peak lists were first merged if they were within 1 bp (H3K4me3 and H3K27Ac) or 200 bp of each other (H3K27me3 and RNAPII). For each merged peak, a single original peak with the lowest FDR was selected as the representative and used in the plot.

## Additional files

Additional file 1: The yield of soluble chromatin is increased in the range of tested temperatures (25 °C -65 °C). Two 20-μm sections from mouse liver FFPE tissues were processed by Chrom-EX PE at indicated temperatures. To accurately measure the impacts of incubation temperature on chromatin yield, we isolated the chromatin by sonication but no MNase treatment after tissue-level cross-linking reversal. DNAs were purified from soluble fraction and insoluble pellet fraction and were quantified using Qubit dsDNA High Sensitivity assay. The percentage of soluble chromatin was calculated from two independent experiments. (PDF 50 kb)

Additional file 2: The genomic location of primer pair along with the peak profiles of H3K4me3 and H3K27me3 marks are visualized for ChIP-seq data generated from frozen and FFPE liver tissues in the Integrative Genomics Viewer. The arrow below the RefGene track indicates the location of primer pair used in the study. The peak profiles indicate the antibodies for H3K4me3 and H3K27me3 marks are specific. (PDF 49 kb)

Additional file 3: DNA profiles for chromatin inputs from 4 liver FFPE tissues relative to Fig. 2a. Two 20-μm sections from mouse liver FFPE tissues were processed by Chrom-EX PE at 65 °C condition. 2.5% input were decross-linked and purified by MinElute PCR Purification Kit and eluted in 16 μl TE and 2 μl DNAs were analyzed by the Fragment Analyzer. (PDF 31 kb)

Additional file 4: DNA profiles in RNAP II, H3K27Ac, H3K4me3 and H3K27me3 ChIP-seq libraries from mouse liver FFPE and frozen tissues. Lane 1, 2: RNAP II libraries from FFPE and frozen tissues; Lane 3–5: H3K27Ac, H3K4me3 and H3K27me3 libraries from FFPE tissues; Lane 6–8: H3K27Ac, H3K4me3 and H3K27me3 libraries from frozen tissues. (PDF 38 kb)

Additional file 5: The overall enrichment of three histone marks and RNA pol II is compared from dataset generated from frozen and FFPE liver samples. The average signal levels of H3K4me3 and H3K27Ac are shown across peak center at the upstream and downstream 2 kb. And the average signal levels of H3K27me3 and RNA Pol II are shown across peak region at the upstream and downstream 2 kb. (PDF 64 kb)

Additional file 6: Summary of mapping results from the libraries generated from frozen and FFPE tissues. (PDF 54 kb)

Additional file 7: Replicates overlapping in mouse frozen liver tissues. (PDF 35 kb)

## Abbreviations

ChIP: Chromatin immunoprecipitation
ChIP-seq: Chromatin immunoprecipitation followed by sequencing
Chrom-EX PE: Chromatin extraction method from FFPE tissues
DNase-seq: DNase I hypersensitive sites sequencing
FFPE: Formalin-fixed paraffin-embedded
FiT-seq: Fixed-tissue chromatin immunoprecipitation sequencing
MNase: Micrococcal nuclease
PAT-ChIP: Pathology tissue-ChIP
RNAP II: RNA polymerase II
TSS: Transcription start site
