# Supplementary figures and images for: Enhanced and controlled chromatin extraction from FFPE tissues and the application to ChIP-seq
Source: BMC Genomics. 2019 Mar 29;20:249. doi: 10.1186/s12864-019-5639-8 (PMC6440302; doi:10.1186/s12864-019-5639-8)

Additional file 1

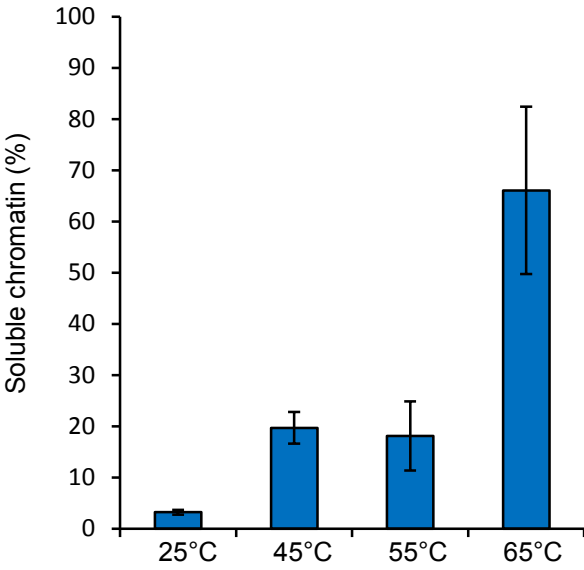

Supplement: Supplementary file 1 — The yield of soluble chromatin is increased in the range of tested temperatures (25 °C -65 °C). Two 20-μm sections from mouse liver FFPE tissues were processed by Chrom-EX PE at indicated temperatures. To accurately measure the impacts of incubation temperature on chromatin yield, we isolated the chromatin by sonication but no MNase treatment after tissue-level cross-linking reversal. DNAs were purified from soluble fraction and insoluble pellet fraction and were quantified using Qubit dsDNA High Sensitivity assay. The percentage of soluble chromatin was calculated from two independent experiments. (PDF 50 kb) [file 12864_2019_5639_MOESM1_ESM.pdf]

Additional file 2

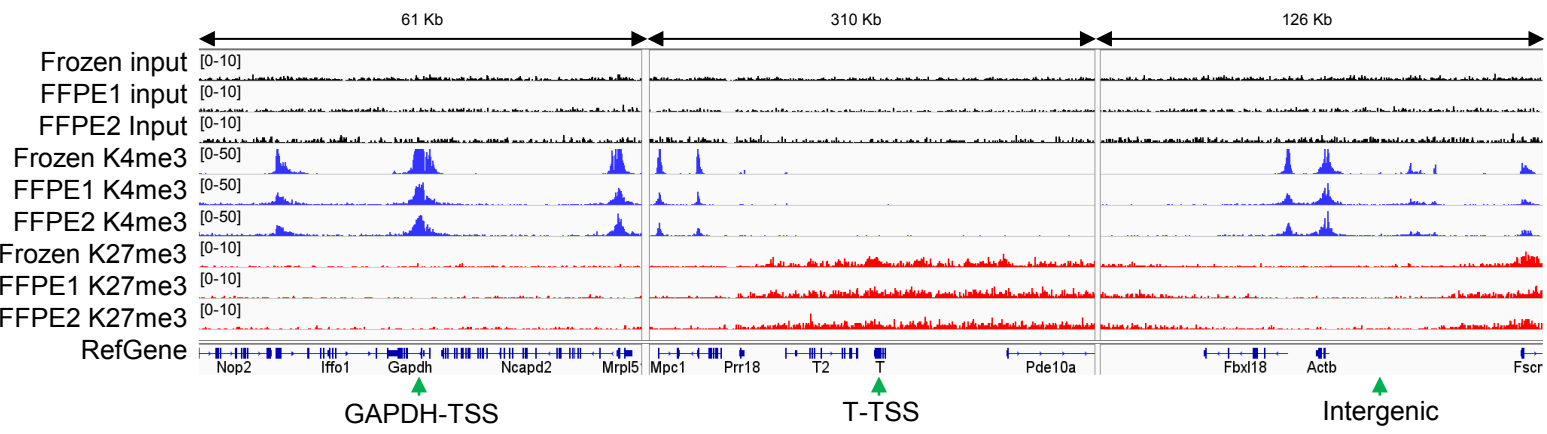

Supplement: Supplementary file 2 — The genomic location of primer pair along with the peak profiles of H3K4me3 and H3K27me3 marks are visualized for ChIP-seq data generated from frozen and FFPE liver tissues in the Integrative Genomics Viewer. The arrow below the RefGene track indicates the location of primer pair used in the study. The peak profiles indicate the antibodies for H3K4me3 and H3K27me3 marks are specific. (PDF 49 kb) [file 12864_2019_5639_MOESM2_ESM.pdf]

Additional file 3

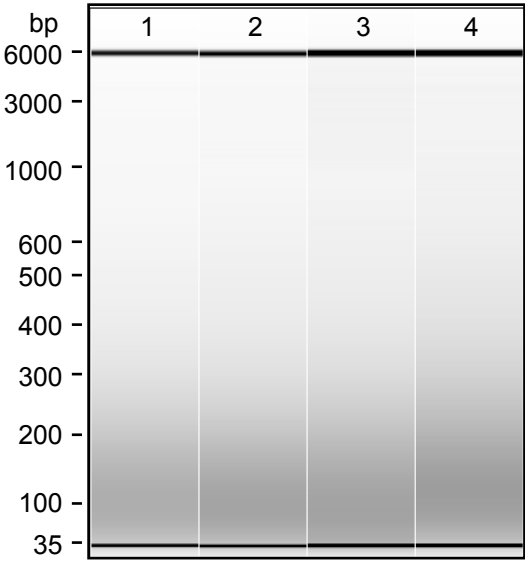

Supplement: Supplementary file 3 — DNA profiles for chromatin inputs from 4 liver FFPE tissues relative to Fig. 2a. Two 20-μm sections from mouse liver FFPE tissues were processed by Chrom-EX PE at 65 °C condition. 2.5% input were decross-linked and purified by MinElute PCR Purification Kit and eluted in 16 μl TE and 2 μl DNAs were analyzed by the Fragment Analyzer. (PDF 31 kb) [file 12864_2019_5639_MOESM3_ESM.pdf]

## Additional file 4

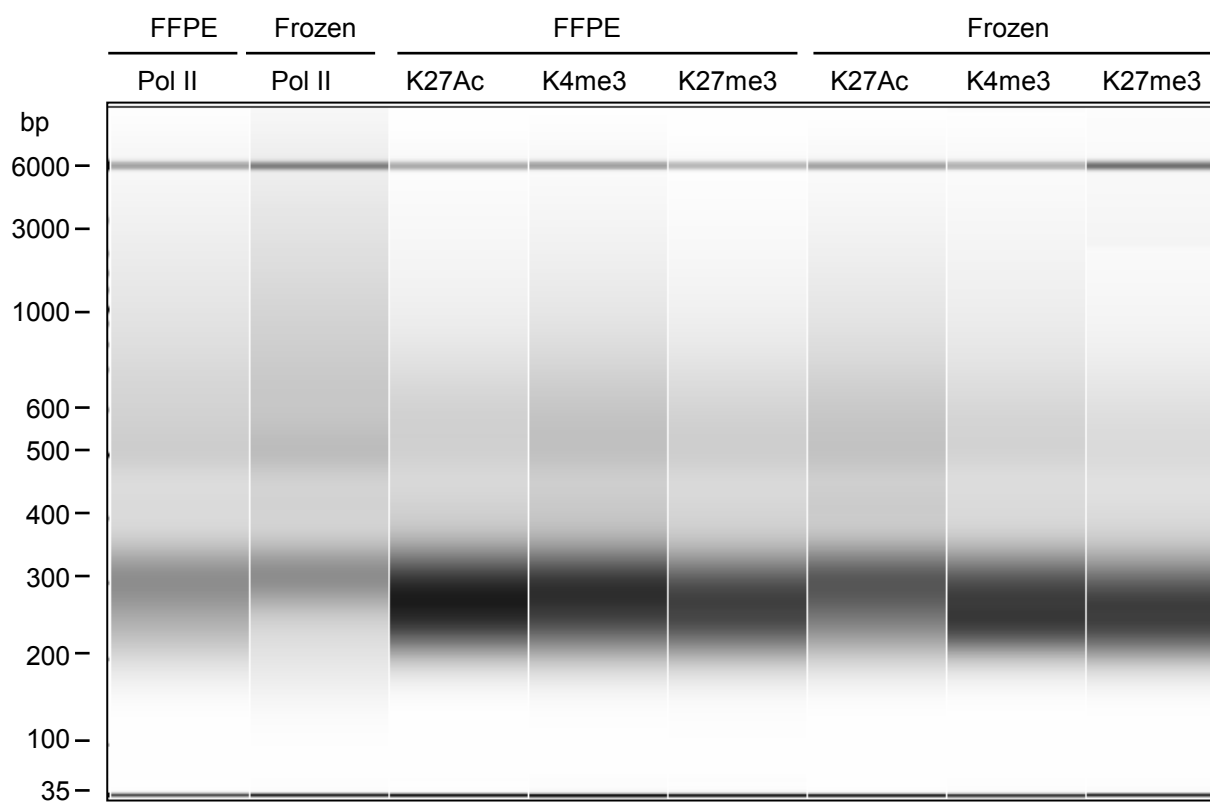

Supplement: Supplementary file 4 — DNA profiles in RNAP II, H3K27Ac, H3K4me3 and H3K27me3 ChIP-seq libraries from mouse liver FFPE and frozen tissues. Lane 1, 2: RNAP II libraries from FFPE and frozen tissues; Lane 3–5: H3K27Ac, H3K4me3 and H3K27me3 libraries from FFPE tissues; Lane 6–8: H3K27Ac, H3K4me3 and H3K27me3 libraries from frozen tissues. (PDF 38 kb) [file 12864_2019_5639_MOESM4_ESM.pdf]

Additional file 5

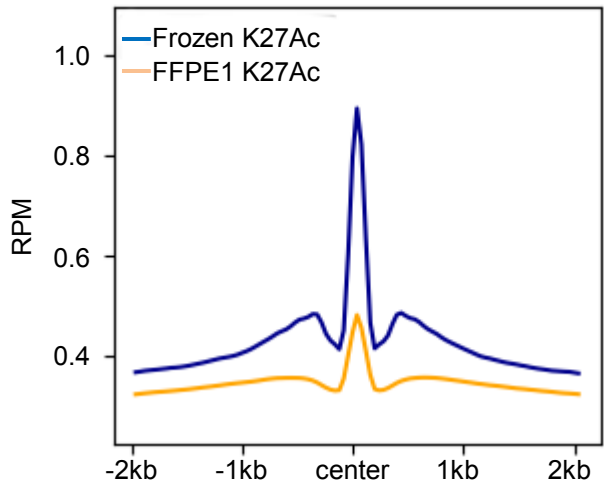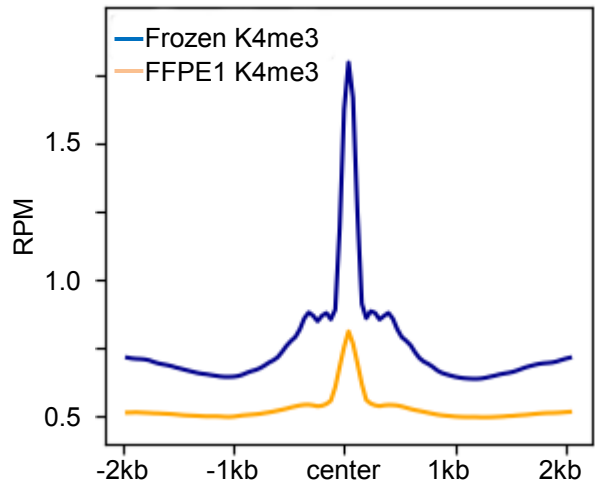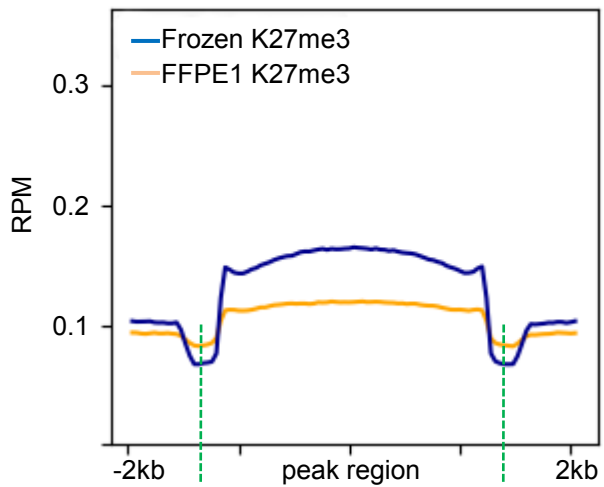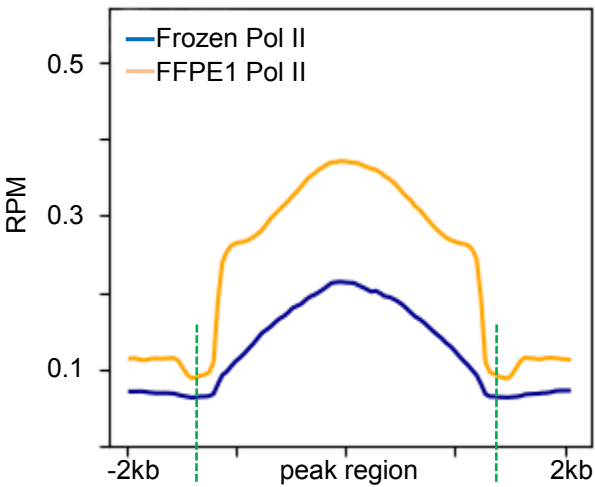

Supplement: Supplementary file 5 — The overall enrichment of three histone marks and RNA pol II is compared from dataset generated from frozen and FFPE liver samples. The average signal levels of H3K4me3 and H3K27Ac are shown across peak center at the upstream and downstream 2 kb. And the average signal levels of H3K27me3 and RNA Pol II are shown across peak region at the upstream and downstream 2 kb. (PDF 64 kb) [file 12864_2019_5639_MOESM5_ESM.pdf]
