# Supplementary material for: Enhanced and controlled chromatin extraction from FFPE tissues and the application to ChIP-seq
Source: BMC Genomics. 2019 Mar 29;20:249. doi: 10.1186/s12864-019-5639-8 (PMC6440302; doi:10.1186/s12864-019-5639-8)
Supplement: Supplementary file 6 — Summary of mapping results from the libraries generated from frozen and FFPE tissues. (PDF 54 kb) [file 12864_2019_5639_MOESM6_ESM.pdf]

Additional file 6

Library Name	Algorithm	Total # of Pairs	Total # Uniquely Mapped Pairs	% Unique Pairs	Complexity	Total Peaks	% Duplicates
Frozen-liver input		39,267,305	28,990,945	73.90%	0.9557		4.43%
Frozen-spleen input		37,224,449	26,924,593	72.30%	0.9550		4.50%
Frozen-liver H3K27Ac	macs	24,840,157	21,559,544	86.80%	0.9546	113,364	4.54%
Frozen-liver H3K4me3-1	macs	53,071,008	46,480,152	87.60%	0.8913	49,441	10.87%
Frozen-liver H3K4me3-2	macs	21,741,804	14,599,613	67.20%	0.9856	13,482	1.44%
Frozen-liver H3K27me3-1	sicer	26,290,236	20,723,733	78.80%	0.9681	30,193	3.19%
Frozen-liver H3K27me3-2	sicer	23,883,876	18,422,609	77.20%	0.9898	21,869	1.02%
Frozen-spleen H3K27Ac	macs	30,470,871	25,943,982	85.20%	0.9449	98,641	5.51%
Frozen-spleen H3K4me3	macs	32,677,576	27,766,238	85.00%	0.9284	52,991	7.16%
Frozen-spleen H3K27me3	sicer	30,946,502	25,456,790	82.30%	0.9585	38,955	4.15%
FFPE-liver input		52,168,890	31,890,133	61.40%	0.9634		3.66%
FFPE-spleen input		50,129,311	30,462,245	60.80%	0.9668		3.32%
FFPE-liver H3K27Ac-1	macs	63,679,779	48,918,941	76.80%	0.6853	128,624	31.47%
FFPE-liver H3K27Ac-2	macs	30,868,956	23,805,686	77.10%	0.4713	92,165	52.87%
FFPE-liver H3K4me3-1	macs	44,069,984	30,725,129	69.70%	0.5560	56,688	44.40%
FFPE-liver H3K4me3-2	macs	31,427,746	22,338,905	71.10%	0.5066	48,129	49.34%
FFPE-liver H3K4me3-3	macs	22,613,053	11,638,381	51.50%	0.9677	21,582	3.23%
FFPE-liver H3K4me3-4	macs	22,141,605	10,418,129	47.10%	0.9785	16,828	2.15%
FFPE-liver H3K27me3-1	sicer	27,777,003	20,687,621	74.50%	0.9350	19,765	6.50%
FFPE-liver H3K27me3-2	sicer	32,498,738	23,949,171	73.70%	0.9479	23,234	5.21%
FFPE-liver H3K27me3-3	sicer	22,926,803	17,788,057	77.60%	0.9813	29,409	1.87%
FFPE-liver H3K27me3-4	sicer	23,171,405	17,851,046	77.00%	0.9821	27,865	1.79%
FFPE-spleen H3K27Ac	macs	32,464,028	24,341,374	75.00%	0.9525	71,648	4.75%
FFPE-spleen H3K4me3	macs	25,206,538	18,598,658	73.80%	0.9514	33,512	4.86%
FFPE-spleen H3K27me3	sicer	23,774,649	17,115,132	72.00%	0.9656	13,332	3.44%
Frozen-liver pol II-1	sicer	20,077,656	15,264,807	76.10%	0.9843	10,843	1.57%
Frozen-liver pol II-2	sicer	25,295,425	19,027,113	75.30%	0.9830	7,874	1.70%
FFPE-liver pol II-1	sicer	19,953,737	12,242,202	61.40%	0.5891	12,825	41.09%
FFPE-liver pol II-2	sicer	22,723,276	13,408,047	59.00%	0.5984	16,421	40.16%
