# Supplementary material for: Enhanced and controlled chromatin extraction from FFPE tissues and the application to ChIP-seq
Source: BMC Genomics. 2019 Mar 29;20:249. doi: 10.1186/s12864-019-5639-8 (PMC6440302; doi:10.1186/s12864-019-5639-8)
Supplement: Supplementary file 7 — Replicates overlapping in mouse frozen liver tissues. (PDF 35 kb) [file 12864_2019_5639_MOESM7_ESM.pdf]

Additional file 7

Replicates overlapping in mouse frozen liver tissues

Mark	Total Peaks 1	Total Peaks 2	% Overlap
H3K4me3	13,482	49,441	99.08%
H3K27me3	21,869	30,193	71.70%
Pol II	10,843	7,874	90.13%
